# Need glucose to sprout: local metabolic control of angiogenesis

**DOI:** 10.1002/emmm.201303174

**Published:** 2013-08-27

**Authors:** Anne Eichmann, Michael Simons

**Affiliations:** 1 Yale Cardiovascular Research Center, Yale University School of MedicineNew Haven, CT, USA; 2 Department of Cellular and Molecular Physiology, Yale University School of MedicineNew Haven, CT, USA; 3 CIRB, Collège de France, Inserm 1050Paris, France; 4 Department of Cell Biology, Yale University School of MedicineNew Haven, CT, USA

**Keywords:** angiogenesis, glycolysis, metabolism

See related article in Cell http://dx.doi.org/10.1016/j.cell.2013.06.037

Much of the focus of research in angiogenesis has been on the molecular mechanisms regulating various critical endothelial cell processes such as migration, proliferation and capillary sprouting. This has led to a number of insights including the key roles played by VEGF signalling in initiating angiogenesis (Chung & Ferrara, 2011; Koch & Claesson-Welsh, 2012) and Notch signalling in regulating its extent via control of these processes (Benedito & Hellstrom, 2013; Cristofaro et al, 2013; Thomas et al, 2013). This further evolved into therapeutic approaches designed to control angiogenesis by regulation of these signalling events that are now understood in a significant amount of detail. Yet little attention however has been paid so far to the metabolic cost of angiogenesis.

All active cellular events, including the ones referred to above, require the expenditure of energy. The underlying assumption has always been that energy (*e.g*. ATP) is abundant and is not a limiting factor as endothelial cells are typically found in the high oxygen environment of blood flow. Indeed, while arterial endothelium is bathed in blood with nearly 100 mm Hg of partial pressure of oxygen (PO_2_), even venous and lymphatic endothelial cells are exposed to a much higher PO_2_ (∼40 mmHg) than most other tissues. Furthermore, essentially all studies of growth factor signalling in vitro that have given us our current understanding of VEGF and Notch biology, have also been carried out in high oxygen environments. Yet in many instances angiogenesis is initiated in low oxygen-environments: consider the developing retina or tumour vasculature where tip cells, an endothelial cell subset that largely is responsible for initiation of new capillary growth, may well be in O_2_-limited environment.

In this context, the recent study of De Bock et al (2013) is especially timely and important. The authors show that endothelial cells, even in a high oxygen environment, generate most of their energy via glycolysis and not via oxidative or fatty acid metabolism. Furthermore, ATP is generated in this fashion throughout the cell including at lamellipodia, which are crucial to capillary tip cell sprouting. Finally, the availability of energy is itself a key factor controlling sprouting: a sufficient supply of energy is enough to overcome the suppressive effect of Notch signalling that hitherto was considered to be the dominant signalling mechanism controlling this event. These observations propel considerations of endothelial metabolism to the centre stage.

**Figure 1:**
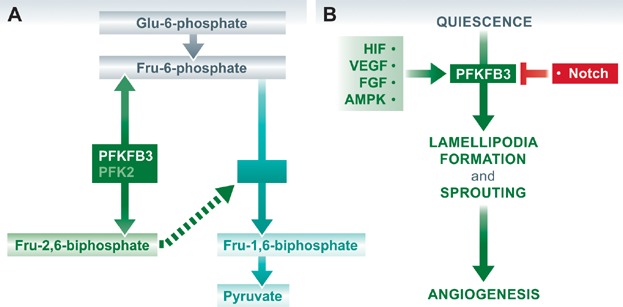
PFKFB3 dependent regulation PFKFB3-dependent regulation of PFK1-dependent conversion of Fru-60-hopsphate to Fru-1,6-bisphosphate.The putative central role played by PFKFB3 in sprouting angiogenesis.

The authors concentrated their attention on the initial key step of glycolysis: conversion of fructose-6-phosphate to fructose-1,6-biphosphate. This is accomplished by the rate-limiting enzyme phosphofructokinase-1 (PFK1). PFK1 activity, in turn, is modulated by an allosteric regulator, fructose-2,6-bisphosphate (Fru-2,6-BP). The latter is also produced from fructose-6-phosphate by another enzyme, 6-phosphofructo-2-kinase/fructose-2,6-bisphosphatase-3 (PFKFB3) also known as phosphofructokinase-2 (PFK2) (Fig 1A).

PFKFB3 is one of four closely related proteins in the PFKFB family and is the predominant isoform expressed in endothelial cells. Interestingly, the expression of the molecule itself is regulated by HIF-1α- (Obach et al, 2004) and AMPK (Mendoza et al, 2012), an essential step in driving endothelial metabolism under energy-limiting circumstances. Endothelial deletion of PFKFB3 resulted in impaired angiogenesis that was characterized by a reduction in endothelial sprouting *in vitro* and *in vivo*. In mosaic sprouting assays the number of PFKFB3-deficient cells was reduced at the tip position. At the cellular level there was a decrease in lamellipodia formation and reduction in the rate of cell migration. In contrast, endothelial overexpression of PFKFB3 increased sprouting, lamellipodia formation and promoted tip position. Most importantly, PFKFB3 overexpression was able to overrule the inhibitory effects of the Notch intracellular domain on sprouting.

The implication of these data is that energy production regulates sprouting and that Notch achieves its sprouting-inhibiting effect in part by decreasing PFKFB3 expression. Notch thus emerges, quite unexpectedly, as a metabolic regulator. Yet PFKFB3-null endothelial cells are not energy starved. They do decrease ATP consumption and reduce protein synthesis and cell proliferation but maintain a number of other cell functions expected of normal quiescent endothelial cells. One implication of this finding is that PFKFB3 levels may regulate endothelial quiescence.

Another interesting observation in this study is that a PFKFB3/PFK1 complex was observed to be associated with the cytoskeleton in lamellipodia. This points to the fact that endothelial cells likely use, and produce energy locally and not globally. This has a number of implications. First, cell migration depends on ATP glycolytically produced at the lamellipodia to organize the cytoskeleton and orchestrate cell movement. This arrangement avoids the high costs of transporting ATP generated elsewhere at the cost of a somewhat less efficient means of production-glycolysis *versus* oxidative phosphorylation. Second, a global assessment of endothelial energy balance may not be all that informative. Instead, one may have to look at energy production associated with a specific cellular activity, *e.g*. sprouting and migration. Third, Notch signalling results in local regulation of energy production. This is achieved, in part, by inhibition of PFKFB3 expression although the details of the pathway remain unknown.

It is also interesting to consider these findings in the broader context of endothelial biology. Recent studies have suggested that one of the key roles of FGF signalling in the endothelium, achieved via activation of MAPK, is maintenance of its normal homeostasis; indeed a reduction in this signalling input results in the loss of vascular integrity, reduction of VEGFR2 expression and endothelial-to-mesenchymal transition (Chen et al, 2012; Murakami et al, 2008). The fact that MAPK activation induces PFKFB3 expression (Bolanos, 2013; Novellasdemunt et al, 2013) is consistent with the key role of glycolysis in normal endothelial energy production. FGF signalling controls normal endothelial function that likely involves a certain level of energy production, which in turn may entail regulation of basal levels of PFKFB3 expression. At the same time, inhibition of FGF signalling dramatically reduces angiogenesis (Bono et al, 2013) which may in part be related to its ability to regulate PFKFB3 levels. In this scheme of things, PFKFB3 might be envisioned as a central regulator of cell sprouting and angiogenesis (Fig 1B).

VEGF-B/Nrp1 is another endothelial signalling system that has been linked to free fatty acid transport and endothelial metabolic regulation. VEGF-B binding to neuropilin-1 (Nrp1) enhances expression of fatty acid transporters thereby increasing fatty acid transport and decreasing glucose uptake (Hagberg et al, 2010). Notch inhibits Nrp1 expression; this may result in suppression of VEGF-induced PFKFB3 expression and down-regulation of fatty acid uptake. This would make Notch a potential key regulator of endothelial metabolism.

In summary, Carmeliet and colleagues made an important advance by identifying a new mechanism controlling endothelial sprouting and angiogenesis. There is much to learn here but the payoffs will likely be very significant.
